# 515 Cognitive Screening After Inpatient Burn Stay to Determine Follow up Needs

**DOI:** 10.1093/jbcr/irae036.150

**Published:** 2024-04-17

**Authors:** Kelsey B Peter, Audrey M O'Neil, Natalie Fitzgerald, Suzanne Totty, Brett C Hartman

**Affiliations:** Richard M Fairbanks Burn Center, Fishers, IN; Richard M. Fairbanks Burn Center, Indianapolis, IN; Richard M. Fairbanks Burn Center at Eskenazi Health, Indianapolis, IN; Indiana University, Indianapolis, IN; Richard M Fairbanks Burn Center, Fishers, IN; Richard M. Fairbanks Burn Center, Indianapolis, IN; Richard M. Fairbanks Burn Center at Eskenazi Health, Indianapolis, IN; Indiana University, Indianapolis, IN; Richard M Fairbanks Burn Center, Fishers, IN; Richard M. Fairbanks Burn Center, Indianapolis, IN; Richard M. Fairbanks Burn Center at Eskenazi Health, Indianapolis, IN; Indiana University, Indianapolis, IN; Richard M Fairbanks Burn Center, Fishers, IN; Richard M. Fairbanks Burn Center, Indianapolis, IN; Richard M. Fairbanks Burn Center at Eskenazi Health, Indianapolis, IN; Indiana University, Indianapolis, IN; Richard M Fairbanks Burn Center, Fishers, IN; Richard M. Fairbanks Burn Center, Indianapolis, IN; Richard M. Fairbanks Burn Center at Eskenazi Health, Indianapolis, IN; Indiana University, Indianapolis, IN

## Abstract

**Introduction:**

Following extended admissions, burn survivors are at risk for continued cognitive impairments at discharge. These impairments are often attributed to environmental factors, poor sleep hygiene, and medications. Despite observing cognitive deficits throughout admission, follow up to determine if impairments resolve after discharge typically does not occur. Individuals have reported extended difficulty with higher executive functioning tasks such as medication management, money management and return to work following ICU admissions, this has not been studied in burn survivors.

**Methods:**

An interdisciplinary cognitive screening process was developed for burn survivors for post-burn ICU discharge. Patients who had a ≥ 14-day admission were screened on the day of discharge and in the outpatient clinic at one-month post-discharge. The Short Blessed Test (SBT) was used as the screening tool with a perfect score being 0 and the worst score being a 28. Patients who demonstrated mild cognitive impairment (SBT score >8) post-discharge were to be referred to outpatient speech therapy for further assessment and treatment.

**Results:**

During the 9-month trial period, 18 patients received the initial discharge screening, with 8 receiving the additional 1 month follow up screen. The 10 patients who did not complete follow up screening were either lost to follow up (n= 9) or declined participation (n=1). At discharge, the average SBT score for all 18 patients was 7.38, ranging from 0 to 22. Discharge locations for these patients included Long-Term Acute Care Hospital (n=4), Acute Rehab (n=5), Subacute Rehab (n=6), and Home (n=3). Overall, 5 of the 10 patients (50%) who missed follow up screening demonstrated cognitive impairment at discharge (Avg 8.0). Average discharge SBT score for the 8 patients who received both screenings was 6.63, ranging from 0 to 15. No patients demonstrated cognitive impairments at the 1 month follow up with average SBT score of 3.4.

**Conclusions:**

Burn survivors are at risk for continued cognitive impairment following burn ICU admissions. Conducting cognitive screening in burn patients is feasible within a verified burn center, and necessary to improve follow up care for burn survivors. Upon evaluation of this process and the results of the screening, it was determined that a new cognitive screen would be beneficial to capture a greater variety of cognitive domains and improve identification of impairments after discharge. The plan for future screening will be to utilize the Montreal Cognitive Assessment Test (MoCA), allowing individuals to be assessed over 8 different domains. The MoCA can also be utilized over the phone, improving patient access to follow up screening.

**Applicability of Research to Practice:**

Cognitive screening of burn patients following ICU admissions has not previously been described in the literature. Little is known about post-burn ICU recovery as it relates to cognitive functioning and long-term deficits.

